# Lipoprotein Ratios: Correlations With Glycated Hemoglobin Among Type 2 Diabetes Mellitus Patients

**DOI:** 10.7759/cureus.53665

**Published:** 2024-02-05

**Authors:** Prabhat LNU, Jagriti LNU, Ayan Banerjee, Akash Bansal, Javin B Gogoi

**Affiliations:** 1 Biochemistry, All India Institute of Medical Sciences Gorakhpur, Gorakhpur, IND; 2 Biochemistry, All India Institute of Medical Sciences Patna, Patna, IND; 3 Biochemistry, Soban Singh Jeena Government Institute of Medical Sciences and Research, Almora, IND

**Keywords:** lipoprotein ratios, tg/hdl-c, type 2 diabetes mellitus, ldl-c/hdl-c ratio, glycated hemoglobin (hba1c)

## Abstract

Introduction

Diabetes mellitus (DM) has become a common disorder in India, and can be even considered as an epidemic in most developing countries. It usually adds a big burden on the economy through its macro and microvascular complications which often require hospitalisation. Glycated hemoglobin (HbA1c) is considered a well-established test to track long-term glycemic control, and hence can be used for both diagnosis and prognosis of disease. On the other hand, lipid profile is a significant marker of cardiovascular risks.

Objective

To investigate the clinical relevance of lipid profile and correlate with glycemic control in type 2 DM patients.

Methodology

This observational study used laboratory results (HbA1c and lipid profile) of 140 patients who attended various out-patient departments (OPD) of All India Institute of Medical Sciences (AIIMS), Gorakhpur. On the advice of clinicians, for routine follow-up, blood samples were collected from the patients (aged 20-50 years, 84 males, and 56 females, with a history of more than three years of type 2 DM). The sera were analyzed for HbA1c and lipid profile [which included triglycerides (TG), total cholesterol (TC), low density lipoprotein cholesterol (LDL-C) and high density lipoprotein cholesterol (HDL-C)]. Based on HbA1c levels the study subjects were divided into three groups, namely group I (HbA1c <7%, n=14), group II (HbA1c7%-8.5%, n=91), and group III (HbA1c >8.5%, n=35). Correlation studies between HbA1c and parameters of lipid profile were explored in the study. Data generated were checked for normality and correlation studies were accordingly done.

Results

Elevated levels of HbA1c were associated with a notable parallel increase in LDL-C levels (P<0.05), TG, and TC. There was no notable correlation observed between HbA1c and HDL-C levels. However, as HbA1c levels increased, the TG/HDL-C and LDL-C/HDL-C ratios displayed a gradual rise (P<0.05).

Conclusion

LDL-C and the LDL-C/HDL-C ratio serve as valuable tools for evaluating and mitigating cardiovascular disease risk and are correlated to glycemic control among individuals with type 2 DM.

## Introduction

Diabetes mellitus (DM) is a common disorder of the current era, which is responsible for many premature deaths. Moreover, it adds a big burden on health infrastructure through its macro and microvascular complications. Major causes of type 2 DM (T2DM) are obesity, improper dietary habits, and fluctuating patterns of lifestyle. According to the World Health Organization (WHO), by the year 2025, 134 million Indians will be suffering from DM. T2DM mostly manifests through insulin resistance in peripheral organs. Besides playing a pivotal role in the metabolism of glucose, insulin also influences the metabolism of lipids.

DM alters lipid profiles to various extents and often manifests as a peculiar form of dyslipidemia. Fatty acid flux to the liver pertains to the release of fatty acids from adipocytes, a process that is negatively regulated by an insulin hormone. The rate of triglyceride (TG) secretion by the liver is insulin-sensitive. So, as the sensitivity to insulin curtails i.e., as the threshold into DM is traversed, the natural inhibitory impact on lipid synthesis by the liver and release of fatty acids from the adipocytes is grossly reduced [1].

The characteristic dyslipidemia in diabetes patients includes hypertriglyceridemia [2], which often results from increased synthesis and decreased clearance of triglyceride-rich lipoproteins (TRL) in fasting and non-fasting states. Moreover, hypertriglyceridemia also implicates high very low-density lipoprotein cholesterol (VLDL-C), as it is the major transporter of TG [3]. In addition, low VLDL clearance, decreased hepatic intake and increased postprandial triglyceride-rich chylomicrons further lead to hypertriglyceridemia [2]. High TG levels have been directly associated with low high-density lipoprotein cholesterol (HDL-C) levels [4] and high low-density lipoprotein cholesterol (LDL-C) levels [5]. Hypertriglyceridemia stimulates the activity of cholesteryl ester transfer protein, which adds TG to HDL leading to its catabolism [6]. However, low HDL-C which was considered as an implication of insulin resistance has been found to further exacerbate abnormal glucose metabolism [7].

Assessment of insulin resistance and cardiovascular risk are cumbersome and costly process requiring specialized tests, which are often not available in remote areas of the country. Lipid profile, which includes TG and HDL-C, is a routine test that is more affordable than even insulin tests. Moreover, lipid profiles and derived parameters have been studied in various populations and are well-standardized. Furthermore, the utility of lipid profile and other routine parameters has been suggested by some of the studies. Our study intends to explore the correlation between lipid profile and glycemic status.

## Materials and methods

Study setting and design

The cross-sectional study included a total of 140 patients with T2DM. The study included individuals visiting medicine out-patient departments (OPDs) at All India Institute of Medical Sciences (AIIMS) Gorakhpur. Prior to initiation of study, ethical clearance from the institute’s ethical board had been obtained with reference no. IHEC/AIIMS-GKP/BMR/120/2023. The study was conducted between June 2022 and July 2022.

Sample size calculation

The sample size was calculated considering 5% Type I error and 80% power (20% type II error) with prevalence of T2DM as 9.3% in the study population. Enrolled subjects were divided into three groups according to their glycated hemoglobin (HbA1c) levels. Participants with HbA1c <7% (N=14) were included in Group I (good glycemic control) and those with HbA1c 7%-8.5% (N=91) were Group II. Group III included individuals with HbA1c >8.5% (N=35).

Inclusion and exclusion criteria

T2DM patients, with at least three years history of disease, coming to medicine OPD at AIIMS Gorakhpur were enrolled in the study. Written and informed consent were obtained before recruitment of subjects in the study.

Patients with type 1 diabetes mellitus, pregnant women, and patients with liver, kidney or muscle diseases were excluded from the study. Individuals with previous history of hyperthyroidism, hypothyroidism, serious infections, and malignancy, or those taking any drugs known to cause disturbance of lipid metabolism were also excluded from the study.

Sampling

Detailed history and relevant general and systemic clinical examination of subjects were conducted. Demographic data, anthropometrics clinical details, family history of diabetes and duration of diabetes were also recorded.

Fasting 3 ml venous blood samples were collected from study subjects. The serum samples separated from whole blood were processed for routine investigations on an automated analyzer Olympus AU400 by colorimetric method. The biochemical parameters done for enrolled subjects were lipid profile (including TC, HDL-C, LDL-C, VLDL-C and TG) and HbA1c.

HbA1c levels were assessed using micro-column chromatography, while TC and TG levels were determined using an enzymatic method, and HDL-C and LDL-C levels were measured using the direct method. Subsequently, TG/HDL-C, TC/HDL-C, and LDL-C/HDL-C ratios were computed.

Statistical analysis

The Statistical Package for Social Sciences version 16 (SPSS Inc., Chicago, IL, USA) was used to code, enter and analyze all statistical data. Descriptive statistical methods were employed to access the data, and the Kolmogorov-Smirnov test was used to determine if the parameters followed a normal distribution. Continuous variables were presented as mean and standard deviation (SD). Pearson’s Correlation and Spearman correlation studies were done for parametric data and non-parametric data respectively. Statistical significance was defined as p-value ≤ 0.05 after analyzing the findings and 95% confidence intervals (CI).

## Results

This observational research utilized laboratory data encompassing HbA1c levels and lipid profiles from a cohort of 140 patients seeking care across diverse OPDs at AIIMS, Gorakhpur. Following clinical recommendations for routine monitoring, blood samples were obtained from individuals aged between 20 and 50 years, comprising 84 males and 56 females, all with a documented history of T2DM spanning over three years. TC, TG, and LDL-C were found to be progressively increasing with higher levels of HbA1c. However, HDL-C did not increase with increased HbA1c levels, with minimum value of 42±11 mg/dl at HbA1c levels of 7-8.5% (Table 1).

**Table 1:** Lipid profile of study subjects. HDL: High Density Lipoprotein, LDL: Low-Density Lipoprotein, HbA1C: Glycated Hemoglobin

Group	Total cholesterol (mg/dl)	Triglyceride (mg/dl)	HDL cholesterol (mg/dl)	LDL Cholesterol (mg/dl)
I (HbA1c <7%)	165±49	145±26	51±14	86±38
II (HbA1c 7-8.5%)	175±56	154±31	42±11	110±47
III (HbA1c >8.5%)	190±63	179±43	46±9	121±54

TC exhibited a gradual increase with rising levels of HbA1c (Table 1). Similarly, LDL-C and TG levels also increased with escalating HbA1c levels (Table 1).

Table 2 reflects the mean±standard deviation values of HbA1c among various groups.

**Table 2:** HbA1c of study subjects HbA1C: Glycated Hemoglobin

Group	HbA1c (%)
I (HbA1c <7%) : N=14	6.7±0.2
II (HbA1c 7-8.5%) : N=91	7.8±0.6
III (HbA1c >8.5%) : N=35	9.6±0.9

A significant correlation was observed between TC and HbA1c (ρ=0.272, P=0.04) (Table 3). In addition, HbA1c displayed a significant correlation with TG (ρ=0.279, P=0.04) (Table 3). Similarly, LDL-C demonstrated a significant correlation with HbA1c (ρ=0.46, P=0.01). Conversely, no significant correlation was found between HbA1c and HDL-C (Table 3). There was also a significant correlation observed between HbA1c and TG/HDL-C ratio (Table 3) (ρ=0.261, P=0.05). However, with increasing levels of HbA1c, both TC/HDL-C (ρ=0.035, P=0.768) and non-HDL/HDL (ρ=0.031, P=0.782) ratios displayed a clear upward trend, with a non-significant correlation (Table 3). Moreover, the LDL-C/HDL-C ratio exhibited a gradual increase and showed a significant correlation with HbA1c (ρ=0.31, P=0.01) (Table 3). Notably, these lipid ratios, particularly the LDL-C/HDL-C ratio, appeared to be more sensitive indicators of impaired lipid metabolism in patients with T2DM.

**Table 3:** Correlation of lipid indices with HbA1c *Correlation is significant at 0.05 (2-tailed) level TG: Triglyceride, HDL-C: High Density Lipoprotein cholesterol, LDL-C: Low-Density Lipoprotein cholesterol, HbA1C: Glycated Hemoglobin

Parameters	Spearman Correlation	
N=140	Spearman correlation coefficient (ρ)	P value
Total Cholesterol	0.272*	0.04
TG	0.279*	0.039
HDL-C	-0.040	0.736
LDL-C	0.46*	0.01
TG / HDL-C	0.261*	0.050
Total Cholesterol / HDL-C	0.035	0.768
Non-HDL-C / HDL-C	0.031	0.782
LDL-C / HDL-C	0.31*	0.01

## Discussion

Several studies have investigated the relationship between HbA1c and lipid profile parameters, aiming to elucidate their interplay in the context of metabolic disorders. A study by Yan et al. demonstrated a positive correlation between HbA1c levels and TC and LDL-C [8]. The same study demonstrated a significant correlation between HbA1c levels and TC/HDL-C and LDL-C/HDL-C ratios. The study further suggests that poorer glycemic control is associated with adverse lipid profile changes, predisposing individuals to increased cardiovascular risk. Moreover, HbA1c has been shown to have a direct correlation with cardiovascular risks [9].

In a cross-sectional retrospective analysis conducted by Sharahili et al. [10], higher HbA1c levels were significantly associated with unfavourable lipid profiles, characterized by elevated LDL-C and TG levels, and reduced HDL-C levels, independent of other metabolic risk factors. The study concluded that HbA1c was significantly associated with TC and TG levels in the T2DM patients.

Conversely, a study by Sarkar et al. reported conflicting results, indicating a weak or non-significant correlation between HbA1c and lipid profile parameters in non-diabetic individuals [11]. However, this study suggests that the relationship between HbA1c and lipid profile may vary depending on the underlying metabolic status of the population studied.

In our study involving diabetic patients, we observed a significant correlation between HbA1c levels and LDL-C, aligning with findings reported by multiple researchers who have also noted significant correlations between HbA1c and parameters of lipid profile [12]. Similarly, we did find significant correlation between HbA1c and TG (and TC). Notably, diabetic patients with high HbA1c demonstrated a notable increase in TG/HDL-C and LDL-C/HDL-C ratios.

Our findings suggest that the regulation of impaired glycemic control, as indicated by HbA1c levels, correlates proportionally with the lipid profile deviations, particularly on the LDL-C/HDL-C ratio. This relationship may be attributed to the fact that changes in these ratios precede alterations in individual lipid levels, especially among patients with initially normal blood lipid profiles. Studies have indicated that patients with T2DM exhibit heightened susceptibility to vascular diseases linked with LDL-C [13].

However, our study could not establish a significant correlation between HDL-C with HbA1c. In contrast, Rader et al. discovered that HDL-C levels were a significant and independent risk factor, exhibiting a stronger association with coronary artery disease (CAD) development compared to TC and LDL-C [14]. Another study similarly found that low HDL-C was a threat for cardiovascular disease (CVD) in older adults, while LDL-C did not demonstrate a significant association with CVD development [15].

Recent evidence suggests that lipid ratios provide greater sensitivity to the severity of coronary heart disease (CHD) compared to individual lipid markers [16]. Blood lipid ratios are more informative than single lipid profile parameters in assessing the outcome of CAD [17]. Similarly, Sun et al. demonstrated that lipid ratios are better than individual lipid parameters in prevention of CHD [18]. Shai et al. reported that TC/HDL-C, LDL-C/HDL-C, and apo B/apo A ratios are more indicative of increased cardiovascular mortality than single lipid parameters, suggesting that these ratios may promote or counteract arteriosclerosis [19].

In summary, the majority of patients with T2DM experience varying degrees of dyslipidemia, which becomes more severe with increasing HbA1c levels. In comparison to individual lipid indices, changes in lipid ratios can detect impaired lipid metabolism at an earlier stage, with the LDL-C/HDL-C ratio emerging as a sensitive indicator. Therefore, the LDL-C/HDL-C ratio proves beneficial in evaluating and mitigating the cardiovascular disease risk associated with impaired lipid metabolism in T2DM. However, further studies with large sample sizes are required to establish the exact relationship among various populations.

Limitations

The major limitation of this study is that it has been conducted in a single center and thus has a small sample size.

## Conclusions

The majority of individuals diagnosed with T2DM encounter varying degrees of dyslipidemia, which tends to worsen as HbA1c levels increase. Our study reflected that TC, LDL and TG increase with HbA1c levels, which is very well in correlation with dyslipidemia associated with T2DM. Moreover, HbA1c was found to have the most sensitive correlation with LDL-C. Derived lipid ratios also provide significant correlation with HbA1c. Additional research involving larger sample sizes is necessary to establish the precise relationship across different populations.
